# Early and mid-term outcomes of thoracic endovascular aortic repair to treat aortic rupture in patients with aneurysms, dissections and trauma

**DOI:** 10.1093/icvts/ivac042

**Published:** 2022-02-15

**Authors:** Julia Benk, Matthias Siepe, Tim Berger, Friedhelm Beyersdorf, Stoyan Kondov, Bartosz Rylski, Martin Czerny, Maximilian Kreibich

**Affiliations:** Department of Cardiovascular Surgery, University Heart Center Freiburg, Faculty of Medicine, Albert-Ludwigs-University of Freiburg, Freiburg, Germany

**Keywords:** Aortic rupture, Thoracic endovascular aortic repair, Aortic dissection, Traumatic aortic injury, Aneurysms

## Abstract

**OBJECTIVES:**

The aim of this study was to analyse outcomes of thoracic endovascular aortic repair to treat aortic rupture.

**METHODS:**

Patient and outcome characteristics of all emergent endovascular treatments for thoracic aortic rupture between January 2009 and December 2019 were analysed.

**RESULTS:**

Thoracic aortic rupture occurred in patients with aortic aneurysms (*n* = 42, 49%), aortic dissection (*n* = 13, 16%) or after trauma (*n* = 30, 35%). Preoperative cerebrospinal fluid drainage was placed in 9 patients (11%) and 18 patients (21%) underwent perioperative supra-aortic transposition. The proximal landing zones were: zone 1 (*n* = 1, 1%), zone 2 (*n* = 23, 27%), zone 3 (*n* = 52, 61%) and zone 4 (*n* = 9, 11%). Temporary spinal cord injury occurred in 1 patient (1%), permanent spinal cord injury in 7 patients (8%). Two patients (2%) experienced a postoperative stroke. Seventeen patients (20%) expired in-hospital. Aortic dissection (odds ratio: 16.246, p = 0.001), aneurysm (odds ratio: 9.090, *P* = 0.003) and preoperative shock (odds ratio: 4.646, *P* < 0.001) were predictive for mortality. Eighteen patients (21%) required a stent-graft-related aortic reintervention for symptomatic supra-aortic malperfusion (*n* = 3, 4%), endoleaks (*n* = 6, 7%), a second aortic rupture (*n* = 4, 5%), retrograde type A aortic dissection (*n* = 2, 2%), aortic-oesophageal fistulation (*n* = 2, 2%) and stent-graft kinking (*n* = 1, 1%).

**CONCLUSIONS:**

Thoracic endovascular aortic repair in patients with aortic rupture has become a valuable treatment modality to stabilize patients. However, a significant risk of postoperative morbidity and mortality remains, particularly in patients with aortic dissections, aneurysms or shock. Patients require thorough follow-up ideally in an aortic clinic with a staff having the entire spectrum of cardiovascular and thoracic surgical expertise.

**Figure:**
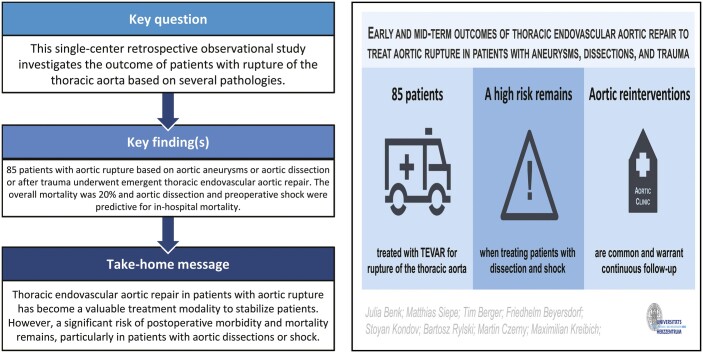

## INTRODUCTION

Rupture of the thoracic aorta is one of the gravest emergencies in vascular surgery. Such critical situations require urgent treatment, ideally in an experienced aortic centre [1]. Open repair of the descending aorta in these patients is associated with high mortality around 20%, and up to 15% of those who survive to suffer from complications like paraplegia, stroke or kidney failure [1–3]. Thoracic endovascular aortic repair (TEVAR) has emerged as a valuable treatment alternative in emergency settings [2, 3]. In fact, provided certain clinical and radiographic conditions, such as a suitable landing zone, are fulfilled and the surgical expertise is available, TEVAR is the therapy of choice in these critical situations [4, 5].

Our aim was to analyse early and follow-up outcomes of emergent TEVAR to treat aortic rupture in a large aortic centre with a dedicated aortic team.

## PATIENTS AND METHODS

### Ethics statement

Our institutional review committee (Albert-Ludwigs-Universität Freiburg, Ethik-Kommission, Prof. Dr R. Korinthenberg) approved this retrospective study (approval number: 20-1302; approval date: 4 February 2021), and the need for informed consent was waived.

### Patients and follow-up protocol

Between January 2009 and December 2019, all patients with the diagnosis of rupture of the descending aorta, who were treated with TEVAR, in a single aortic centre were included in this study. We selected all patients for TEVAR if suitable landing zones and appropriate access vessels were fulfilled and the surgical expertise was available. The aortic rupture was defined as disruption of all the layers of the aortic wall [6]. Our routine follow-up protocol includes visits to our dedicated aortic clinic after 6, 12 months and yearly thereafter. All patients underwent computed tomography angiography scans to confirm their acute aortic diagnosis, before discharge, at every follow-up visit and when clinically warranted.

### Aortic repair approach

Following diagnostic computed tomography angiography, patients were transferred immediately to the operating room. In case of traumatic aortic rupture and other serious injuries, surgical treatment was coordinated interdisciplinary with other essential disciplines including traumatology and neuro-surgery. Cerebralspinal fluid (CSF) drainages were placed for spinal cord protection in stable patients whenever feasible. Contraindications were oral anticoagulation therapy and unstable haemodynamics. If the landing zone for the stent graft was 2 and the patient was haemodynamically stable, subclavian-to-carotid transposition or bypass was performed either before or immediately after TEVAR. In case of zone 1 TEVAR, double transposition was performed before TEVAR. We always aimed for a ‘healthy’ landing zone in trauma and aneurysm patients and intended for 15% oversizing. In dissection patients, we aimed for proximal oversizing in the ‘healthy’ landing zone of 10% and we did not oversize distally. All endovascular procedures were performed via femoral access. In the early study period, an open cut down was applied. More recently, percutaneous access using pre-closure devices has become the first choice for femoral access.

### Outcome measures

Data were collected retrospectively using our prospectively maintained aortic database. The primary outcome was in-hospital mortality and secondary outcomes were stroke, spinal cord injury, acute kidney failure and need for reintervention. The modified Rankin Scale (mRS) was used to classify the severity of a postoperative stroke [7]. All strokes were assessed by consulting neurologists. Non-disabling strokes were defined as postoperative strokes causing no clinical symptoms (mRS 0), no significant disability (mRS 1) or slight disability (mRS 2). The rest was classified as disabling strokes. Spinal cord ischaemia was evaluated by standardized clinical examination [8]. Postoperative CSF drainage was placed in all symptomatic patients that were too unstable for preoperative placement. In case paraplegia was completely reversible following CSF drainage placement, spinal cord ischaemia was classified as temporary.

### Statistical analysis

Data are presented as absolute and relative frequency or as median [first quartile, third quartile] and are compared among patients with the underlying disease aneurysm, dissection and trauma. Analysis of variance was used for normally distributed data and the Kruskal–Wallis test was used for non-normally distributed data. Normality was assessed using the Kolmogorov–Smirnov test. Categorical variables were compared using the Chi-squared test with the calculation of exact values. In case of small group sizes (*n* < 5), Fisher’s exact test was used. A value of *P* < 0.05 was considered statistically significant. Survival was analysed and compared using the Kaplan–Meier method and log-rank test. A Cox regression model with clinically selected variables (sex, age, ruptured aneurysm, ruptured dissection, intubation on arrival, shock on arrival) was performed to identify factors associated with overall mortality. A competing risk analysis was performed to analyse the influence of selected variables (age, sex, aneurysm, dissection) on the risk for reintervention. To compute risk estimates and confidence intervals for 12, 24 and 36 months, we used a cubic smoothing spline. This addresses scarcity of observations that causes purely non-parametric techniques to suffer from high variance. Smoothing ensures the estimates are more robust and stable. A *P*-value of <0.05 was considered to represent a statistically significant difference. Statistical analysis was conducted using IBM SPSS Statistic 21.0 (IBM-SPSS Inc., Armonk, NY, USA) and R version 3.5.1 (The R Foundation for Statistical Computing, Vienna, Austria). Statistical analysis was conducted using IBM SPSS Statistic 21.0 (IBM-SPSS Inc.).

## RESULTS

### Patient characteristics

All demographics and risk factors of the 85 patients are summarized in Table 1. Forty-two patients (49%) were admitted with rupture of an aortic aneurysm, 13 patients (16%) presented with an aortic rupture of an acute (*n* = 10, 12%) or chronic (*n* = 3, 4%) aortic dissection and 30 patients (35%) suffered a traumatic penetration of the descending aorta. Patients with aneurysms and dissections were older and presented with a higher incidence of cardiovascular risk factors and comorbidities than younger patients suffering traumatic rupture. On admission, 26 patients (31%) were already intubated. In total, 25 patients (29%) presented in haemorrhagic shock, defined as dependence on catecholamine or continuous intravenous fluid therapy. Forty per cent of the patients with traumatic rupture, 46% of the patients with dissection and 17% with a ruptured aneurysm. The incidences varied significantly among the 3 groups.

**Table 1:** Patient characteristics

	All patients	Aneurysm	Dissection	Trauma	*P*-value
	(*n* = 85)	(*n* = 42)	(*n* = 13)	(*n* = 30)	
Age (years)	67 [57, 75]	68 [59, 75]	68 [56, 75]	63 [48, 73]	<0.001
Male	52 (61)	27 (64)	6 (46)	19 (63)	0.520
Diabetes mellitus type 2	12 (14)	9 (21)	1 (8)	2 (7)	0.207
Hyperlipidaemia	12 (14)	10 (24)	0 (0)	2 (7)	0.048
Hypertension	37 (44)	27 (64)	8 (62)	2 (7)	<0.001
History of smoking	12 (14)	9 (21)	1 (8)	2 (7)	0.207
COPD	4 (5)	4 (10)	0 (0)	0 (0)	0.204
History of stroke	2 (2)	2 (5)	0 (0)	0 (0)	0.647
Chronic renal impairment	15 (18)	15 (36)	0 (0)	0 (0)	<0.001
Dialysis	2 (2)	2 (5)	0 (0)	0 (0)	0.647
Connective tissue disease	1 (1)	0 (0)	1 (8)	0 (0)	0.153
Coronary artery disease	15 (18)	13 (31)	2 (15)	0 (0)	0.001

Clinical presentation	All patients	Aneurysm	Dissection	Trauma	*P*-value
	(*n* = 85)	(*n* = 42)	(*n* = 13)	(*n* = 30)	
Dyspnoea	12 (14)	9 (21)	2 (15)	1 (3)	0.093
Pain	58 (68)	34 (81)	12 (92)	12 (40)	<0.001
Intubated pre-hospital	26 (31)	3 (7)	4 (31)	19 (63)	<0.001
Haematothorax	59 (69)	27 (64)	11 (85)	21 (70)	0.402
Shock	25 (29)	7 (17)	6 (46)	12 (40)	0.036
Malperfusion			2 (15)		

Values are *n* (%) or median [fist quartile, third quartile]. Haematothorax: mediastinal haematoma formation, Hounsfield units of pleural effusion >30; Shock, shock, defined as dependence on catecholamine or continuous intravenous fluid therapy.

COPD: chronic obstructive pulmonary disease.

### Operative details

Operative details are summarized in Table 2. In total, 115 stent grafts were implanted, ranging from 1 to 4 devices per patient. More stent grafts were required in patients with aneurysms and dissections than in patients with traumatic aortic injury. The different types of stent-grafts we used are listed in Supplementary Material, Table S1 and the diameter of stent-grafts is listed in Table 2. Zone 3 was the most common proximal landing zone in all patients, while zone 2 was significantly more common in patients with traumatic aortic injury. The stent grafts implanted in patients with ruptured aneurysms and dissections were larger than those implanted for traumatic penetration of the descending aorta.

**Table 2:** Operative details

	All patients	Aneurysm	Dissection	Trauma	*P*-value
	(*n* = 85)	(*n* = 42)	*n* = 13)	(*n* = 30)	
Supra-aortic transposition	18 (21)	7 (17)	2 (15)	9 (30)	0.367
Preoperative CSF drainage	9 (11)	4 (10)	1 (8)	4 (13)	0.893
1 stent graft	55 (65)	28 (67)	4 (31)	23 (77)	0.015
2 stent grafts	22 (26)	9 (21)	6 (46)	7 (23)	0.204
3 stent grafts	7 (8)	4 (10)	3 (23)	0 (0)	0.032
4 stent grafts	1 (1)	1 (2)	0 (0)	0 (0)	1.000
Proximal landing zones					
Zone 1	1 (1)	1 (2)	0 (0)	0 (0)	1.000
Zone 2	23 (27)	7 (17)	3 (23)	13 (43)	0.044
Zone 3	52 (61)	25 (60)	10 (77)	17 (57)	0.454
Zone 4	9 (11)	9 (21)	0 (0)	0 (0)	0.005
Proximal diameter of first stent graft	33 [28, 38]	37 [32, 40]	35 [33, 42]	25 [21, 31]	<0.001
Distal diameter of last stent graft	32 [28, 40]	37 [32, 41]	36 [28, 44]	27 [22, 29]	<0.001

Endoleak perioperative	All patients	Aneurysm	Dissection	Trauma	*P*-value
	(n = 85)	(n = 42)	(n = 13)	(n = 30)	
Ia	2 (2)	0 (0)	1 (8)	1(3)	0.122
Ib	1 (1)	1 (2)	0 (0)	0 (0)	1.000
II	0 (0)	0 (0)	0 (0)	0 (0)	–
III	0 (0)	0 (0)	0 (0)	0 (0)	–
X-ray time in min	8.6 [5.6, 12.4]	9.6 [5.6, 13.7]	12.2 [7.9, 14.5]	7.4 [5.3, 9.5]	0.027
Procedure time in min	95 [68, 133]	100 [77, 128]	95 [64, 154]	92 [68, 134]	0.972

Values are *n* (%) or median [fist quartile, third quartile].

CSF: cerebrospinal fluid.

Immediate technical success was defined as sealing the aortic rupture without endoleak and the absence of malposition or migration of the stent graft or conversion to open repair. In this study, the overall immediate success rate was 97%, for aneurysms 98%, for dissections 92% and 97% for traumatic rupture. There was no statistical relevant difference between the 3 groups. In 3 patients (3%), with stable haemodynamics following TEVAR implantation, a perioperative endoleak was tolerated (Table 2). Two patients with endoleak type Ia required carotico-subclavian bypass and implantation of a second stent graft after 1 week and 1 year, respectively. The third patient suffering from a metastasizing carcinoma had an endoleak type Ib in his huge ruptured aneurysm and was deemed unfit for conversion to open surgery. He expired the following day.

### In-hospital outcome characteristics and aortic-related reinterventions

Seventeen patients (20%) expired in-hospital, 2 (2%) of them on the operating table. Of those 2, the first one presented in a frail state with a ruptured aneurysm and we attempted salvage TEVAR, but ultimately failed to stabilize the patient. The second patient presented with an aortic rupture and fulminant mitral regurgitation due to traumatic papillary muscle rupture after multiple traumas, the haemodynamic situation failed to stabilize after TEVAR. Causes of death of the remaining 15 patients were multi-organ failure (*n* = 8), aortic rupture (thoracic: *n* = 4, abdominal: *n* = 2) and cerebral oedema following a motorcycle accident (*n* = 1). The 2 patients with abdominal aortic ruptures became symptomatic with a large untreated infrarenal aneurysms (60 mm) and a large penetrating abdominal aortic ulcer 1 and 3 days following TEVAR. In all 6 patients with aortic ruptures, consent for further treatment was denied or patients were deemed unfit for conversion to open surgery. The incidence of in-hospital death differed significantly and was higher in patients with dissections and lowest in patients with traumatic aortic injury (*P* = 0.025). Two patients (2%) suffered a disabling stroke (discharge mRS: 4 and 6, respectively). One of them presented initially with syncope and a dissected left carotid artery, while the other revealed no preoperative neurological symptoms. Seven patients (8%) developed permanent spinal cord injury, paraplegia in 1 patient (1%) was reversible after placing CSF drainage. Four of these patients arrived in shock in the operating room. All outcome characteristics are listed in Table 3.

**Table 3:** Outcome characteristics

	All patients	Aneurysm	Dissection	Trauma	*P*-value
	(*n* = 85)	(*n* = 42)	(*n* = 13)	(*n* = 30)	
In-hospital mortality	17 (20)	8 (19)	6 (46)	3 (10)	0.025
Aortic rupture	6 (100)	4 (67)	2 (33)	0(0)	
Multi-organ failure	9 (100)	4 (44)	4 (44)	1 (11)	
Other	2 (100)	0(0)	0(0)	2 (100)	
Non-disabling stroke	0 (0)	0 (0)	0 (0)	0 (0)	–
Disabling stroke	2 (2)	1 (2)	1 (8)	0 (0)	0.371
Temporary spinal cord injury	1 (1)	1 (2)	0 (0)	0 (0)	1.000
Permanent spinal cord injury	7 (8)	5 (12)	1 (8)	1(3)	0.549
Acute kidney failure	6 (7)	2 (5)	2 (15)	2 (7)	0.274
Thoracic drainage	32 (38)	10 (23)	6 (46)	16 (53)	0.021
Tracheotomy	20 (24)	8 (19)	2 (15)	10 (33)	0.255
Intensive care unit stay (day)	6 [3, 11]	4 [2, 8]	4 [3, 9]	10 [5, 14]	0.006

Values are *n* (%) or median [fist quartile, third quartile].

In total, 18 patients (21%) required a TEVAR-related reintervention [aneurysms: *n* = 12 (29%), dissection: *n* = 3 (23%), trauma: *n* = 3 (10%); *P* = 0.16]. During the same hospital stay, 4 patients (5%) required open aortic repair: Two patients (2%) developed a type A dissection post-TEVAR after 2 and 14 days, respectively. Both were successfully treated surgically by replacing the ascending aorta. Another patient (1%) underwent ascending and hemiarch aortic replacement 1 day after TEVAR as a staged approach to tackle an unresolved aneurysm (diameter > 60 mm) that had not been treated in the emergency situation. Another patient (1%) developed an aorto-oesophageal fistulation 2 months after TEVAR: She underwent complete surgical removal of the prosthetic material, orthotopic reconstruction with pericardial tube grafts, extensive local debridement and oesophageal resection. In addition, 2 patients (2%) developed paresthesia of the left arm; they both underwent supra-aortic transposition 2 days after TEVAR, while 4 patients underwent an additional TEVAR procedure because of endoleaks (Type Ia, Ib, II, and III). A stent was placed in the subclavian artery of 1 patient to resolve a flow impairment following TEVAR.

### Cox regression analysis

Our model identified shock on admission (odds ratio: 4.464, *P* < 0.001) and aortic rupture caused by dissection (odds ratio: 16.246, *P* = 0.001) or aneurysm (odds ratio: 9.090, *P* = 0.003) as predictors for overall mortality. All variables in our model are listed in Table 4.

**Table 4:** Cox model: mortality

Variable	HR	95% CI	*P*-value
Age (years)	0.986	0.959–1.019	0.402
Male gender	1.323	0.584–2.999	0.503
Aneurysm	9.090	2.130–38.782	0.003
Dissection	16.246	3.184–82.899	0.001
Intubated	0.854	0.300–2.429	0.768
Shock	4.646	1.946–10.978	<0.001

CI: confidence interval; HR: hazard ratio.

### Long-term follow-up and aortic-related reinterventions

**Figure 1:**
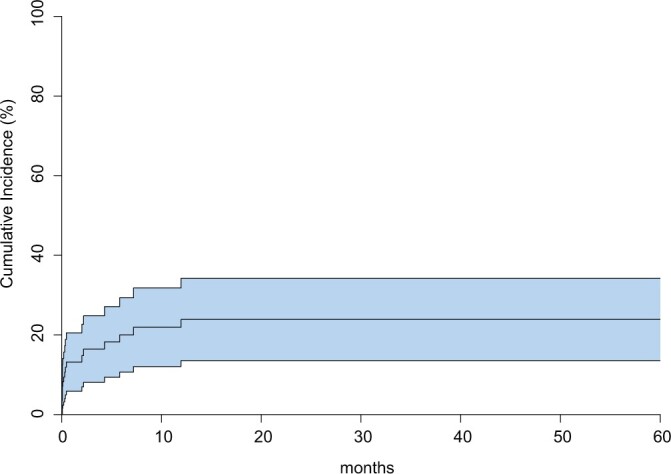
Competing−risks regression for reintervention.

**Figure 2:**
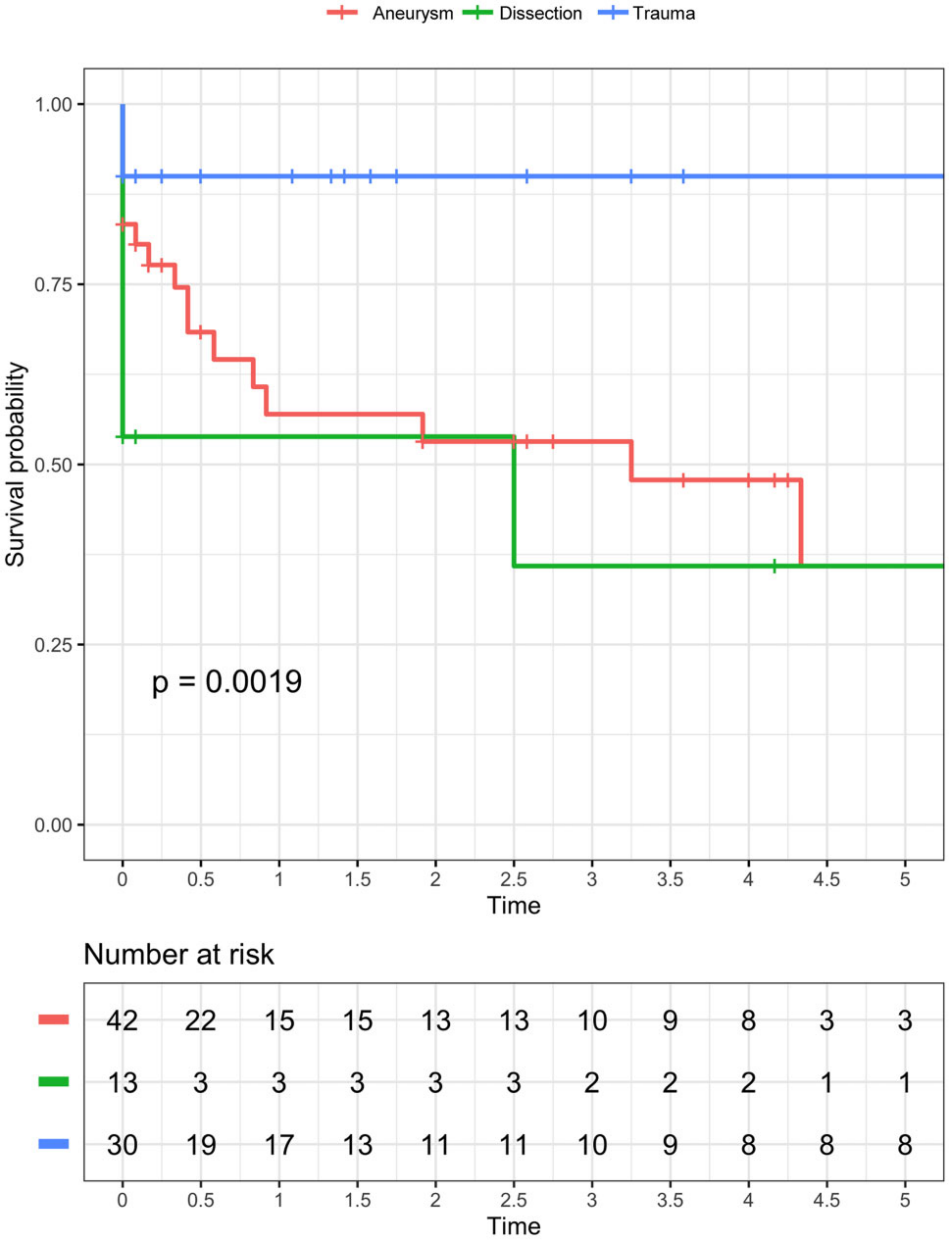
Kaplan–Meier curves depicting late survival in patient who were treated with emergent thoracic endovascular aortic repair secondary to an aortic rupture of an aneurysmatic aorta (red), dissected aorta (green) or after thoracic trauma (blue). Log-rank test: *P* = 0.002.

After discharge, 7 patients required additional TEVAR. Median time to re-TEVAR was 175 [97, 289] days. Indications were: second aortic rupture (*n* = 3), endoleaks type Ib and II (*n* = 2), aorto-oesophageal fistulation (*n* = 1) and stent-graft kinking (*n* = 1). No risk factors for reinterventions were identified in the competing risk model (Table 5). However, the risk for reintervention was 23% (95% CI: 13–33%), 24% (95% CI: 13–35%) and 25% (95% CI: 14–36%) after 12, 24 and 36 months respectively (Fig. 1). Patients were followed up for a total of 155 patient-years, with a median follow-up of 6 [first quartile: 0, third quartile: 36] months. During follow-up, 11 patients expired because of cardiorespiratory failure *n* = 5 (45%), sepsis *n* = 4 (36%), cerebral bleeding *n* = 1 (9%) and 1 (9%) of unknown cause. Late survival is depicted in Fig. 2 and differed significantly among the 3 groups (log-rank: *P* = 0.002).

**Figure 3:**
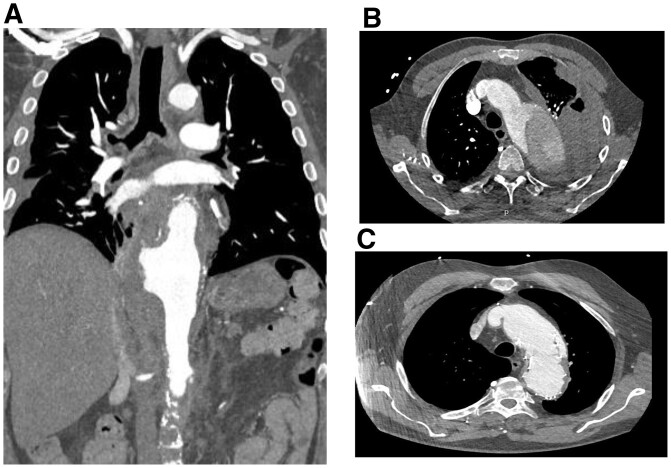
Representative computed tomography angiography scans depicting an aortic rupture (**A**), a haematothorax caused by an acute aortic rupture (**B**) and an endoleak type Ia 4 years after emergent thoracic endovascular aortic repair (**C**).

Representative computed tomography angiography scans depicting an aortic rupture are pictured in Fig. 3.

**Table 5:** Competing risk regression: reintervention^a^

Variable	*P*-value	SHR	95% CI
Age	0.15	0.971	0.989–1.07
Sex	0.69	1.207	0.328–2.09
Aneurysm	0.50	0.581	0.357–8.31
Dissection	0.61	0.612	0.246–10.86

Values are *n* (%).

a Competing risk: death.

CI: confidence interval; SHR: subdistribution hazard ratio.

## DISCUSSION

This study’s key findings can be summarized as: (i) TEVAR in patients with aortic rupture has become a promising treatment modality to stabilize patients, but (ii) a significant risk of postoperative morbidity and mortality remains, particularly in patients with aortic dissections or preoperative shock and (iii) even after initial stabilization, a persistent need for aortic reinterventions remains, underlining how important thorough follow-up is, ideally in an aortic clinic possessing full cardiovascular and thoracic surgical expertise.

In this study, traumatic aortic injury patients were younger, not unusual in trauma patient populations [3, 9–11]. There is evidence that up to 90% of all traumatic aortic injury patients expire at the accident location [12, 13]. Hence, younger, fitter patients with fewer comorbidities are more likely to survive transport to the hospital. This fact is made obvious by the very low incidence of cardiovascular risk factors in this study’s traumatic aortic-injury patients. Moreover, in case of traumatic aortic injury, the thorax trauma is usually accompanied by a frail chest and urgent need for early intubation **[**13**]**, thus the high incidence of intubated patients in this group. On the other hand, patients suffering dissections and aneurysms usually also have significant comorbidities, as in this study [3, 9].

While landing zones 1 and 2 were used in 24 patients, only 18 patients underwent perioperative supra-aortic transposition—a fact possibly attributable to the operation’s urgency and high incidence of preoperative shock on arrival. Obviously, in patients undergoing zone 1 TEVAR, double transposition is mandatory, yet left subclavian artery revascularization can be postponed in case of zone 2 TEVAR [14]. Nevertheless, it is our standard policy to perform concomitant subclavian-to-carotid bypass or transposition in case of zone 2 TEVAR whenever possible, thereby adhering to the most recent recommendations published by the large European societies—not just to preserve brachial arterial blood flow but also to ensure sufficient spinal cord and cerebellar perfusion [5, 15]. Alternatively, endovascular techniques for left subclavian artery revascularization may be promising options with low rates of mortality and major neurologic morbidity even though they were not used in this study [16, 17]. In addition, it is our common practice to place CSF drainage routinely before elective TEVAR to maximize spinal cord protection whenever possible. However, because of the emergency settings and/or oral anticoagulation therapy, in this study, only 9 patients (11%) underwent preoperative CSF drainage placement. This low incidence is a potential explanation for the permanent spinal cord injury in 7 patients, who were all operated without CSF drainage. In these emergent cases, we routinely take other spinal cord protection approaches including elevating the arterial blood pressure, assuring an adequate haemoglobin level, fast-track concept and serial postoperative neurological examination [5, 15]. However, while we routinely try to elevate the arterial blood pressure in these patients following TEVAR in the absence of a periprocedural endoleak, note that 6 patients expired in-hospital because of a ruptured aorta. Hence, the risks and benefits of different spinal cord protection strategies need to be considered carefully for each patient. On the other hand, our 8% permanent spinal cord injury incidence in an emergency setting compares well to reported rates between 1% and 10% in elective surgical situations [15, 18–20]. Stroke is another important neurological complication after TEVAR. The in-hospital stroke rate of 2% in this study is similar to other cohorts [21, 22].

The significant difference in the proximal landing zone and in the stent-graft number needed also reflects the various underlying pathologies well. In general, we look for a non-dissected, non-ectatic ‘healthy’ aortic segment ideally 2.5-cm long, to achieve long-term durability [5]. In case of traumatic injury, the rupture’s location is usually distal to the left subclavian artery at the level of the aortic isthmus [12, 23]. Because these patients usually have no concomitant aortic pathologies, zone 2 or even zone 3 TEVAR will usually suffice to seal off the local rupture site. In addition, because of the localized disease process, the landing zones can be closer to the rupture in these patients. Conversely, in patients with aneurysmatic or dissected aortas, our approach commonly causes proximalization of the landing zone close to or into the aortic arch, accompanied by the need for subclavian-to-carotid transposition or bypass, or even double transposition (which we try to generally avoid in dissection patients). Hence, more stent grafts are required and more aortic segments are generally covered in these patients. Obviously, in an emergent setting with haemodynamically instable patients, a less ideal proximal landing zone may be acceptable to stabilize the patient. Nevertheless, in these patients especially, a risk of recurring aortic events such as a retrograde aortic dissection or aortic rupture remains. Hence, in stable patients without significant comorbidities, we follow the recent European recommendations and also evaluate patients with aortic ruptures for frozen elephant trunk implantation to enable a durable proximal aortic repair [5, 24, 25]. Moreover, because of the high incidence of early and late aortic-related complications in patients with aneurysms and dissections, we tend to more liberally evaluate these patients for a more durable proximal repair (i.e. the frozen elephant trunk repair) after initial TEVAR stabilization, today. After all, the more extensive endovascular strategy in these patients may also be responsible for their worse clinical outcome, particularly in dissections patients.

On admission, around 70% of our patients revealed a haematothorax in computed tomography angiography, while the rest presented with contained ruptures. A thoracic drainage was placed when the patient was suffering from respiratory and/or cardiovascular impairment. This was necessary in 54% of the cases. The thoracic drainage was always placed after treatment of the aortic rupture with successful TEVAR. Although this approach has been reported to lower postoperative morbidity and mortality [10], a quarter of these patients required postoperative tracheotomy in our study.

There is evidence of up to 30% perioperative mortality after a thoracic aorta rupture treated by TEVAR [2, 10, 26]. Our study patients revealed an in-hospital mortality of 20%. However, postoperative mortality is always closely associated with the patients’ preoperative condition. In fact, we observed a high incidence of preoperative shock in our all-comers cohort, and shock proved to be highly predictive for death. In addition, while early mortality was highest in patients with aortic dissection, patients with dissections and aneurysms had similar late survival rates in this study due to their chronically diseased aorta and comparably high incidence of cardiovascular risk factors. Conversely, patients suffering traumatic aortic injury experienced no late death because the aorta is usually not chronically diseased.

Lastly, we identified a significant risk of secondary aortic reinterventions in our competing risk regression model, highlighting the need for continuous aortic follow-up, ideally in a dedicated aortic clinic. Lastly, 2 patients (2%) developed an aorto-oesophageal fistulation during follow-up; the risk for fistula development is known to be higher in conjunction with emergency TEVAR [27]. Our standardized, integrated patient management consists of broad-spectrum antibiotic treatment, complete removal of any prosthetic material, extensive local debridement and orthotopic aortic reconstruction using a self-made xenopericardial graft, which one of the latter 2 patients received [27, 28]. Conversely, the other patient had to undergo emergent re-TEVAR as palliative treatment, and she was discharged alive.

After thoracic aortic rupture therapy via TEVAR, close follow-up in an aortic clinic—ideally with a continuously available and broad spectrum of expert cardiovascular and thoracic treatments—is essential to ensure a good long-term outcome. As this study makes evident, reinterventions after emergency TEVAR are frequent, and both endovascular and open surgical expertise is required to ensure optimum patient-centred care.

### Limitations and strengths

This study is limited by its retrospective nature, the relatively brief follow-up period and the small sample size. One of the main limitations of the analysis is that the 3 groups are very heterogeneous, and, in addition, the sample size is small. Therefore, it is very difficult to adjust the effect of the group (aneurysm/dissection/trauma) on mortality for possible confounders. However, it contributes valuable data on TEVAR in patients who suffer acute rupture of the descending thoracic aorta and highlights the need for their continuous and thorough aortic follow-up.

## CONCLUSIONS

TEVAR has proven to be a good treatment modality to stabilize patients suffering a rupture of the thoracic aorta. However, a significant risk of postoperative morbidity and mortality remains, particularly in patients with aortic dissections or preoperative shock. Moreover, even after initial stabilization and discharge, the need for aortic reinterventions remains, underlying how important it is that these patients be thoroughly followed-up, ideally in an aortic clinic with vast cardiovascular and thoracic surgical expertise.

## SUPPLEMENTARY MATERIAL

Supplementary material is available at *ICVTS* online.

**Conflict of interest:** Martin Czerny is a consultant to Terumo Aortic and Medtronic, received speaking honoraria from Bentley and Cryolife and is shareholder of TEVAR Ltd. Bartosz Rylski is a consultant to Terumo Aortic. Maximilian Kreibich has received speaking honoraria from Terumo Aortic.

## Data availability statement

All relevant data are within the manuscript and its Supporting Information files.

## Author contributions

**Julia Benk:** Conceptualization; Writing—original draft. **Matthias Siepe:** Conceptualization; Formal analysis; Investigation; Supervision; Validation. **Tim Berger:** Conceptualization; Data curation; Investigation; Validation. **Friedhelm Beyersdorf:** Conceptualization; Formal analysis; Investigation; Supervision; Validation. **Stoyan Kondov:** Conceptualization; Data curation; Investigation; Supervision; Validation; Visualization. **Bartosz Rylski:** Conceptualization; Data curation; Supervision; Validation. **Martin Czerny:** Conceptualization; Formal analysis; Supervision; Validation; Writing—review & editing. **Maximilian Kreibich:** Conceptualization; Data curation; Formal analysis; Methodology; Supervision; Validation; Visualization; Writing—review & editing.

## Reviewer information

Interactive CardioVascular and Thoracic Surgery thanks Sven Peterss, Mario Giovanni Gerardo D'Oria and the other anonymous reviewers for their contribution to the peer review process of this article.

## Supplementary Material

ivac042_Supplementary_Data

## ABBREVIATIONS

CSF: Cerebrospinal fluid
mRS: Modified Rankin Scale
TEVAR: Thoracic endovascular aortic repair
