# Supplementary material for: Early and mid-term outcomes of thoracic endovascular aortic repair to treat aortic rupture in patients with aneurysms, dissections and trauma
Source: Interact Cardiovasc Thorac Surg. 2022 Feb 15;35(1):ivac042. doi: 10.1093/icvts/ivac042 (PMC9714596; doi:10.1093/icvts/ivac042)
Supplement: ivac042_Supplementary_Data [file ivac042_supplementary_data.docx]

**Supplemental** **Table 1: Type of stent-grafts**

| Stent-grafts in total | 116 (100) |
| --- | --- |
| Terumo Aortic RELAY® | 60 (52) |
| Medtronic Valiant® | 38 (33) |
| MedtronicEndurant II® | 11 (10) |
| Medtronic Talent® | 2 (2) |
| Gore TAG® | 2 (2) |
| Jotec® | 2 (2) |

Values are n (%)
